# Neighbourhood and path-based greenspace in three European countries: associations with objective physical activity

**DOI:** 10.1186/s12889-021-10259-0

**Published:** 2021-02-04

**Authors:** William Mueller, Paul Wilkinson, James Milner, Sotiris Vardoulakis, Susanne Steinle, Juha Pärkkä, Eija Parmes, Luc Cluitmans, Eelco Kuijpers, Anjoeka Pronk, Denis Sarigiannis, Spyros Karakitsios, Dimitris Chapizanis, Thomas Maggos, Asimina Stamatelopoulou, Miranda Loh

**Affiliations:** 1 Institute of Occupational Medicine, Edinburgh, UK; 2 London School of Hygiene & Tropical Medicine, London, UK; 3 National Centre for Epidemiology and Population Health, Australian National University, Canberra, Australia; 4 VTT Technical Research Centre of Finland, Finland; 5 TNO, Netherlands; 6 Aristotle University of Thessaloniki, Thessaloniki, Greece; 7 National Centre for Scientific Research ‘Demokritos’, Athens, Greece

**Keywords:** Greenspace, Physical activity, Exposure, Walking, Cycling

## Abstract

**Background:**

Greenspace has been associated with health benefits in many contexts. An important pathway may be through outdoor physical activity. We use a novel approach to examine the link between greenspace microenvironments and outdoor physical activity levels in the HEALS study conducted in Edinburgh (UK), the Netherlands, and Athens and Thessaloniki (Greece).

**Methods:**

Using physical activity tracker recordings, 118 HEALS participants with young children were classified with regard to daily minutes of moderate to vigorous physical activity (MVPA); 60 were classified with regard to the metabolic equivalent task (MET)-minutes for each of the 1014 active trips they made. Greenspace indicators were generated for Normalised Difference Vegetation Index (NDVI), tree cover density (TCD), and green land use (GLU). We employed linear mixed-effects models to analyse (1) daily MVPA in relation to greenspace within 300 m and 1000 m of residential addresses and (2) trip MET-minutes in relation to average greenspace within a 50 m buffer of walking/cycling routes. Models were adjusted for activity, walkability, bluespace, age, sex, car ownership, dog ownership, season, weekday/weekend day, and local meteorology.

**Results:**

There was no clear association between MVPA-minutes and any residential greenspace measure. For example, in fully adjusted models, a 10 percentage point increase in NDVI within 300 m of home was associated with a daily increase of 1.14 (95% CI − 0.41 to 2.70) minutes of MVPA. However, we did find evidence to indicate greenspace markers were positively linked to intensity and duration of activity: in fully adjusted models, 10 percentage point increases in trip NDVI, TCD, and GLU were associated with increases of 10.4 (95% CI: 4.43 to 16.4), 10.6 (95% CI: 4.96 to 16.3), and 3.36 (95% CI: 0.00 to 6.72) MET-minutes, respectively. The magnitude of associations with greenspace tended to be greater for cycling.

**Conclusions:**

More strenuous or longer walking and cycling trips occurred in environments with more greenspace, but levels of residential greenspace did not have a clear link with outdoor MVPA. To build on our research, we suggest future work examine larger, more diverse populations and investigate the influence of greenspace for trip purpose and route preference.

**Supplementary Information:**

The online version contains supplementary material available at 10.1186/s12889-021-10259-0.

## Background

Increased residential greenspace (e.g., parks) or greenness (e.g., street trees) has shown to be associated with beneficial health, such as better self-reported health and reduced all-cause and cardiovascular mortality [55]. Research has now progressed to explore potential causal mechanisms. As strong links have been made between physical activity (PA) and numerous health outcomes, particularly for cardiovascular outcomes [59], an important pathway to health may be access to areas in which to engage in PA. Moreover, though still an active research area, exercise specifically undertaken in green areas may enhance the proven benefits of PA [46].

Nevertheless, research on the importance of greenspace for exercise has produced mixed results. Cross-sectional studies relying on self-reported data to assess the relationship between residential greenspace and PA identified positive associations in populations in Australia [2], Canada [35], and the US [52], while other work in Denmark [44], Netherlands [33], and Scotland [37] found no such links. With the emergence of low-cost GPS-equipped sensors and devices [32], researchers can now better track objective measures of PA and actual greenspace use, though these studies too have found equivocal results: the amount of residential greenspace was related to higher levels of overall moderate to vigorous PA (MVPA) [23], but in another study, associations were found only with PA when undertaken within green areas (i.e., not overall PA) [53].

Recommendations from agencies, including the World Health Organization (WHO), prescribe a minimum weekly dose of 150 min of moderate intensity or 75 min of vigorous PA, yet a recent global survey found over a quarter of individuals were not achieving these salubrious levels [18]. Though greenspace may help promote active travel and facilitate outdoor PA, for example, through appealing tree-lined streets or accessible parks, other neighbourhood attributes, such as overall walkability (e.g., street connectivity, population density, mixed use development) and access to services, have been found to be more important [14, 22]. Even if a positive link with greenspace is established, a further complicating factor is that self-selection may bias findings if healthier individuals choose to live in greener areas with more options for outdoor exercise [10]; if present, this bias would result in exaggerated health benefits of greenspace.

Our study explored two distinct research questions to advance our understanding of the association of greenspace and PA within the built environment: 1) whether the availability of residential greenspace is associated with increased MVPA and 2) whether individuals choose routes with on average higher greenspace levels for longer/more active journeys. In addition, for the second question, we also assessed the greenspace associations separately for walking and cycling trips.

## Methods

### Study design and population

Data were obtained from the EU-funded study, Health and Environment-wide Associations based on Large population Surveys (HEALS; http://www.heals-eu.eu), which employed indoor and personal sensors to characterise the environments of families with young children. The study included a sample of households concentrated in Edinburgh, UK; Utrecht and elsewhere in the Netherlands; and Thessaloniki and Athens, Greece. Individuals aged 18 years or older with a young child (< 3 years of age) were eligible to participate in the HEALS study (*n* = 131) and were recruited through advertising via universities, childcare groups, and word of mouth. Informed written consent was provided by all participants. Personal monitoring periods lasted approximately 1 week during 2015 and 2016 and entailed indoor monitoring of air pollutants and noise and the participant wearing a physical activity tracker device. Questionnaires were developed in the HEALS study to gather household data, including socioeconomic position (SEP) (see supplementary material).

### Greenspace

**Fig. 1:**
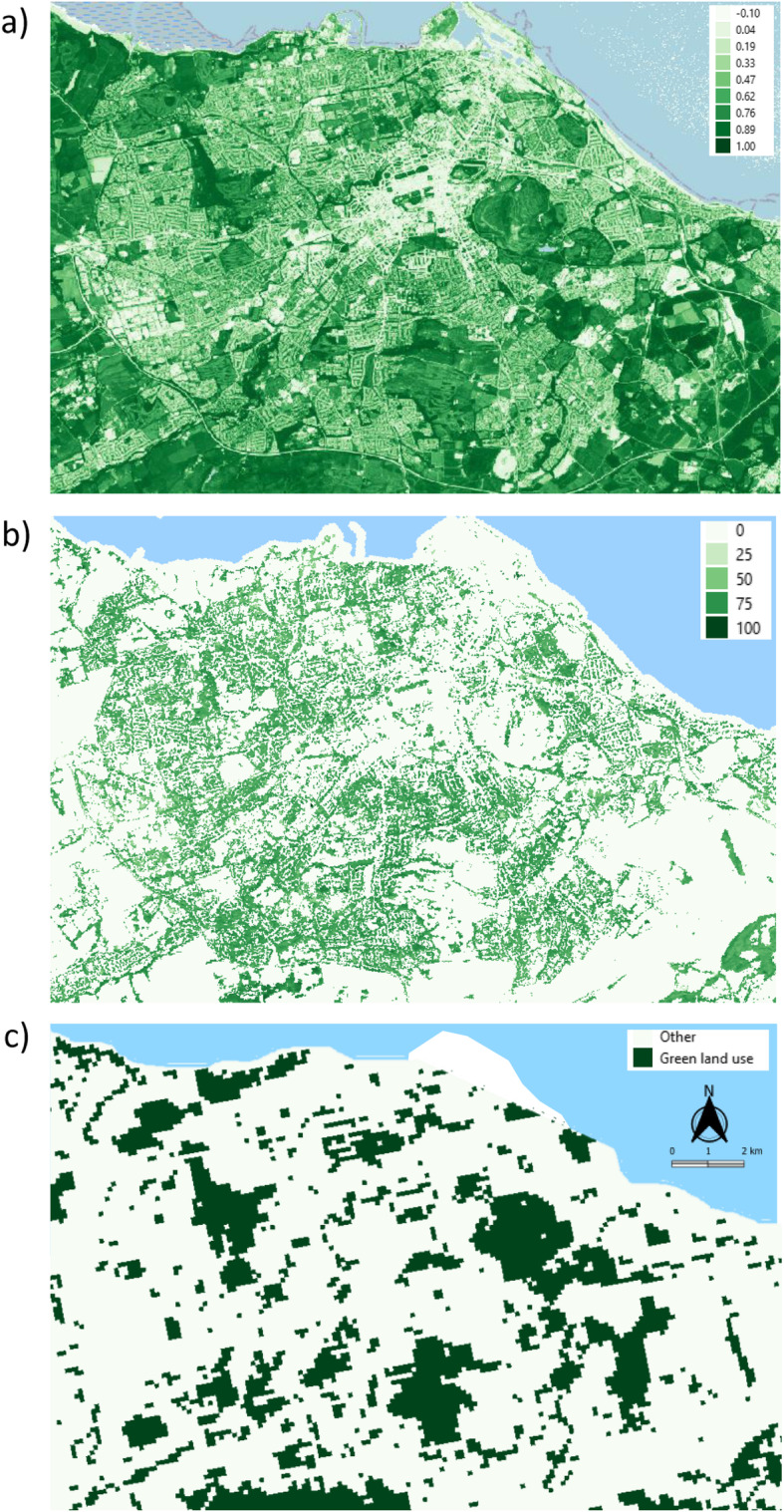
Maps of Edinburgh, UK to illustrate **a** Normalised Difference Vegetation Index (− 0.1 to 1.0), **b** tree cover density (0–100%), and **c** green land use. Basemap from©OpenStreetMap contributors (www.openstreetmap.org), available under the Open Database License

We assigned three indicators of urban greenspace: the Normalised Difference Vegetation Index (NDVI), tree cover density (TCD), and green land use (GLU), similar to a previous analysis using the HEALS dataset published by the authors [36]. Each indicator provides potentially overlapping, but distinct, perspectives of greenspace: NDVI (− 1 to + 1) represents the overall greenness of a given area, TCD provides the percentage (0–100%) of an area covered by the canopy of trees as visible from satellites, and GLU indicates areas used for specific types of green land (parks, forests, sports pitches, etc.) (see Fig. 1).

For each study area, NDVI values were calculated using Sentinel-2 satellite images available from the Copernicus Open Access Hub at 10-m spatial and five-day temporal resolutions. NDVI raster data with values of <− 0.1 represent water or ice and were excluded from greenness calculations [15]. Images from summer with cloud coverage of < 10% were selected to maximise spatial contrasts of greenness. Images produced within 1 year of the personal monitoring periods were retrieved, except for those in and around Edinburgh, due to cloud coverage (See Table S1 for exact image dates). Average annual TCD based on Sentinel-2 and Landsat 8 satellite images (20 m spatial resolution) for Europe in 2015 was also obtained from the Copernicus Hub. Coastal waters were excluded in the calculation of TCD values. GLU was based on CORINE land use data (2012), which has been refined subsequently through data fusion with other spatial datasets (e.g., Urban Atlas, OpenStreet Map) and is publicly available as a 100 m raster dataset [40]. Unlike the original CORINE dataset, this enhanced version distinguishes between green and non-green sport and leisure facilities. The following categories were combined to create a GLU map: green urban areas, green sport and leisure facilities, broad-leaved forest, coniferous forest, mixed forest, natural grasslands, moors and heathland, sclerophyllous vegetation, and transitional woodland-shrub. Mean values of NDVI and TCD, and the proportion of GLU, were calculated in 300 m and 1000 m radial buffers around home addresses. These sizes were selected to represent a reasonable walking distance to greenspace (300 m; [56]) and to reflect a larger, neighbourhood scale (1000 m; [3]). Additional details of the methods for each indicator can be found in Mueller et al. [36].

### Physical activity

During the personal monitoring periods, study participants wore a Fitbit flex device (original version) on their wrist (Fitbit Inc., San Francisco, CA, USA) [12] and installed the ‘Moves’ app (moves-app.com) [13] on their mobile phones; participants were asked to keep their Fitbit and phone with them whenever possible. Fitbits recorded the total number of steps completed each minute and the Moves app recorded GPS locations and the duration, distance, and activity (i.e., walking, running, cycling, vehicle transport) based on its algorithm to identify discrete trips. The Fitbit flex has been found to reliably record steps compared to gold standards (Optogait system and ActivePAL device) [28], and the Moves app can correctly record the location and type of separate trips [4, 47].

To take advantage of both the physical activity sensor and mobile phone app deployed in the HEALS study, we derived two PA metrics that made use of the particular data provided by each sensor: daily minutes of MVPA steps (Fitbit) and Metabolic Equivalent Task minutes (MET-minutes) (Moves app); METs represent the energy cost of an activity relative to a resting state [1]. Daily steps were calculated by summing minutes with ≥100 steps as recorded by the Fitbit flex (equivalent to ≥3 METs) [41] across the monitoring period. These daily values were then divided by the number of days with at least 12 h of data (i.e., 75% complete data, assuming 8 h of sleep), where at least four such days had been recorded during the monitoring period. Out of 133 individuals who were provided Fitbits (some households had multiple participants), 124 (93%) provided sufficient data for analysis.

MET-minutes were calculated by assigning a specific MET to those trips identified by the Moves app to be ‘walking’, ‘running’, or ‘cycling’, depending on the activity; average speed (based on distance and duration, as recorded by Moves); and overall grade change (steepness) during each trip using values set out in Ainsworth et al. [1]. To account for steepness in the calculation of METs, topographical GIS maps (30 m resolution) were acquired from the Japan Aerospace Exploration Agency, based on the Advanced Land Observing Satellite (ALOS-2; [50]). Where no METs were specified by Ainsworth et al. [1] for a given combination of activity/speed/grade, values were interpolated or extrapolated (*n* = 3) (see Table S2 for a complete list of METs used in analysis). METs were multiplied by the duration of each trip to calculate MET-minutes. GPS points were converted to lines in QGIS v.3.10.1 [39] and visual inspection was used to remove trips either with straight lines that did not appear to follow road networks or that traversed bodies of water (*n* = 16). Only six trips were assigned as ‘running,’ which were subsequently excluded from analysis. Values above five standard deviations (SD) in excess of the mean were excluded for MET-minutes (*n* = 7) and duration (*n* = 2). To select trips that occurred outdoors, those of < 3 min in duration or < 100 m in distance were excluded from analysis. As with the daily steps calculation, Moves data were used only from individuals with at least 18 h (i.e., 75%) of complete data on four or more days during the monitoring period. Out of 123 individuals who downloaded Moves onto their phones, 69 (56%) provided sufficient data for analysis. Since few (*n* = 4) participants in Thessaloniki generated sufficient Moves data, this study centre was excluded from the trip-based analysis.

### Walkability

As certain features of the built environment may be more likely to encourage physical activity [14], we calculated walk scores to capture the degree of walkability of residential and travel environments. Similar to previous studies (e.g., [19, 22, 57]), walk scores were calculated based on GIS data using three factors: population density, intersection counts, and land use mix. As well as walking, these same built environment factors may also encourage cycling [26]. Population density was based on global 1 × 1 km gridded estimates for 2015 [7]. Intersection counts were calculated using QGIS via road networks from OpenStreetMap shapefiles downloaded during March–April 2019 from Geofabrik (https://download.geofabrik.de/). Auto-oriented (i.e., non-pedestrian accessible) roads were removed by deleting feature classes for ‘motorway’, ‘service’, or ‘trunk’, and the processing tool in QGIS, ‘v.clean’, was employed to identify intersections of two or more distinct roads. Land use mix was based on the refined CORINE dataset, including ‘commercial/service facilities’, ‘public facilities’, and ‘sport and leisure green/built-up’. Z-scores of each walk score (i.e., mean population density, total intersection counts, and presence of specific land uses) were calculated across all home addresses for the 300 m and 1000 m buffers and were summed to create a walk score. Walk scores calculated separately within and across study areas were highly correlated for both 300 m (*r* = 0.88) and 1000 m (*r* = 0.91) buffers; the latter metric was used for analysis.

To examine the association of greenspace and MET-minutes between different trips taken by the same individual, linear buffers of 50 m were generated for each trip for which mean values of NDVI and TCD, as well as the proportion of GLU, were calculated; a smaller buffer size has been shown to be most strongly associated with MVPA [21]. To account for different trip distances, the number of intersections within each trip buffer was divided by the total distance, which was then used to calculate walk scores in a similar fashion as described above.

### Other covariates

As well as walkability, we adjusted for bluespace, daily meteorology, and season as other environmental factors. We accounted for bluespace by identifying any bodies of water in residential and trip buffers, as bluespace has been shown to be positively correlated with physical activity, especially walking [16, 38]. We included in our definition of bluespace the following CORINE land cover types: ‘water courses’, ‘water bodies’, ‘coastal lagoons’, ‘estuaries’, and ‘sea and ocean’. We obtained for the dates of the personal monitoring periods weather data, including daily maximum temperature and wind speed, and total precipitation from the US National Centers for Environmental Information [27] from the following stations (latitude, longitude): Edinburgh Royal Botanic Garden (55.967, − 3.210); Schiphol, Netherlands (52.316, 4.790); and Hellinikon, Greece (37.900, 23.750). Season was assigned to each monitoring period based on the majority of dates that occurred in a given season. As noted above, during the monitoring periods, participants also completed questionnaires on SEP and other information, including employment status (e.g., working, in school, caring for family), highest education completed, car ownership, and household pets.

### Statistical analysis

We used mixed regression methods to examine associations between greenspace and physical activity metrics. Each greenspace metric (mean NDVI score, mean TCD, and proportion of GLU,) was rescaled such that regression coefficients represented the change in outcome for a 10 percentage point increase in the relevant parameter, an approach adopted by Mueller et al. [36].

Models were developed to assess:

(i) the *between-individuals* association of MVPA with residential greenspace (seeking to answer the question of whether people living in greener areas have higher levels of MVPA),

- and

(ii) the association, *within individuals*, of MET-minutes with trip-based greenspace (seeking to answer the question of whether longer/more active journeys are undertaken in areas with more greenspace compared with shorter/less active journeys).

For (i), with daily MVPA-minutes as the outcome, regression models with a random intercept for study centre were separately developed for residential greenspace metric at 300 m and 1000 m buffers around the home. Model results are presented with various levels of pre-specified confounder adjustment: (1) an unadjusted model, (2) a model adjusted for age using cubic splines with three knots, sex, season, and bluespace (any), and (3) a model with additional adjustment for car ownership, dog ownership, walk score, education, and employment.

For (ii), regression models with random intercepts for both study centre and individual and robust standard errors were separately developed for each of the three greenspace metrics: NDVI, TCD, and GLU. Results are again presented with adjustment for different sets of pre-specified confounders: (1) an unadjusted model, (2) a model with adjustment for age, sex, season, and bluespace (any), and (3) a model with additional adjustments for education, employment status, walk score, day of week, weather conditions on the day of activity, mean residential greenspace (1000 m buffer), car ownership, and dog ownership. Effect modification by activity (i.e., walking and cycling) was examined by including in the models an interaction term between greenspace metric and activity. Cubic splines were included into the model for age and temperature. Geospatial analysis was performed using QGIS and statistical analysis was undertaken using Stata v15 [48].

## Results

A total of 131 households enrolled in the HEALS study across the four study centres, with personal monitoring periods spanning from March 2015 to June 2016. There were 118 and 60 individuals who provided sufficient data and for whom covariate data were available in the neighbourhood and trip-based greenspace analyses, respectively. Descriptive characteristics pertaining to those individuals are presented in Table 1. The mean duration of MVPA-minutes was just under 12 min per day, with a maximum of nearly 40 min. The number of trips recorded for each participant ranged from one to 96, with a mean of 30.3 (SD = 23.8); the mean trip duration was just over 9 min. There was a total of 1014 trips, of which 676 (66.7%) were walking and 338 (33.3%) cycling; 89.9% (*n* = 304) of the cycling trips were in the Netherlands. The mean METs for each trip was 3.8; when accounting for duration, mean MET-minutes equated to 37.0.

**Table 1:** Descriptive characteristics of the study participants

Characteristics	Mean (SD) or N (%)	
	Neighbourhood Greenspace (***n*** = 118)	Trip-based Greenspace (***n*** = 60)
Age (years)	35.0 (5.1)	34.8 (4.0)
Sex		
Male	43 (36.4%)	20 (33.3%)
Female	75 (63.6%)	40 (66.7%)
Daily MVPA-minutes	11.9 (9.8)	–
METs	–	3.8 (1.3)
MET-minutes	–	37.0 (39.0)
Duration (minutes)	–	9.3 (7.7)
Valid data days	–	6.5 (2.9)
Walk score		
300 m residential	−0.02 (2.31)	
1000 m residential	−0.04 (2.34)	
50 m trip-based		0.02 (1.86)
Study Centre Participants		
Athens	25 (21.2%)	20 (33.3%)
Edinburgh	26 (22.0%)	11 (18.3%)
Thessaloniki	23 (19.5%)	0 (0.0%)
Utrecht	44 (37.3%)	29 (48.3%)
Car owner	104 (88.1%)	58 (96.7%)
Dog owner	5 (4.2%)	4 (6.7%)
Season monitored		
Winter	13 (11.0%)	4 (6.4%)
Spring	39 (33.1%)	15 (23.8%)
Summer	49 (41.5%)	35 (55.6%)
Autumn	17 (14.4%)	9 (14.3%)
University educated	88 (74.6%)	54 (90.0%)
Employed	93 (78.8%)	52 (86.7%)
Any bluespace	13 (11.0%)	19 (31.7%)
NDVI (−0.1 to 1.0)		
300 m residential	0.31 (0.16)	
1000 m residential	0.35 (0.18)	
50 m trip-based	–	0.27 (0.15)
TCD (Percentage)		
300 m residential	10.5 (10.4)	
1000 m residential	11.7 (11.2)	
50 m trip-based	–	9.2 (10.6)
GLU (Proportion)		
300 m residential	0.07 (0.11)	
1000 m residential	0.13 (0.13)	
50 m trip-based	–	0.08 (0.16)
Meteorological factors		
Temperature (°C)	–	22.1 (6.6)
Days with rain	–	2.0 (4.5)
Wind speed (knots)	–	13.5 (5.7)

Mean residential greenspace values were slightly higher for the 1000 m compared to the 300 m buffer (Table 1). The average trip-based NDVI was 0.27, with minimum and maximum values of − 0.04 and 0.83, respectively. Trip-based TCD levels ranged from 0 to 73.5%, with 85.5% (*n* = 864) of trips containing tree cover. The percentage of trips with any GLU was 31.2% (*n* = 316), with three (0.3%) trips occurring entirely in places of GLU. The greenspace metrics were weakly to moderately correlated, with NDVI and TCD consistently having the strongest associations. Greenspace metrics were mostly negatively correlated with walk score. There was little apparent correlation between residential greenspace metrics and daily MVPA-minutes. By contrast, trip-based greenspace was moderately correlated with MET-minutes, with coefficient values ranging from 0.44 (GLU) to 0.59 (TCD) (Table 2).

**Table 2:**
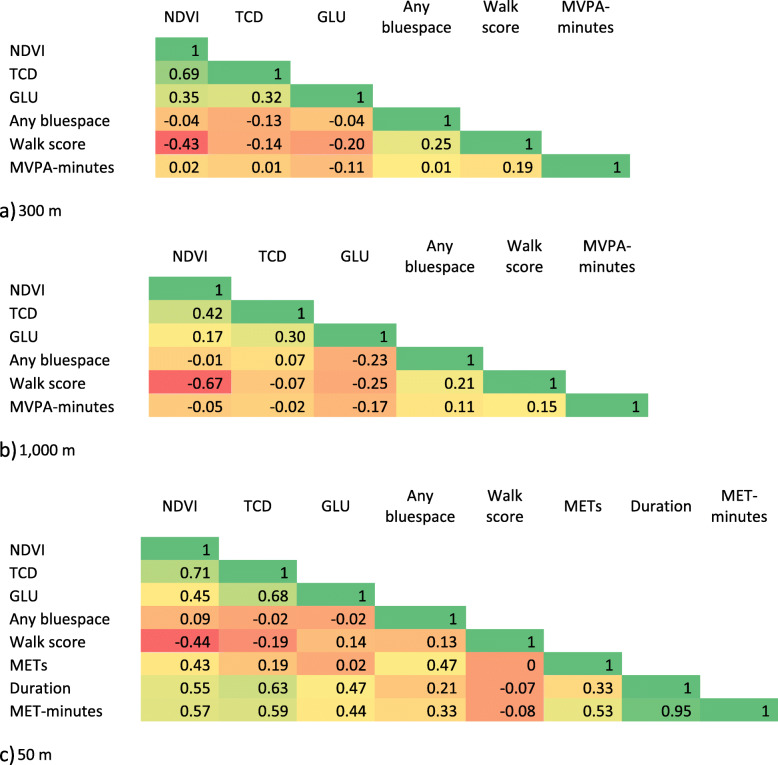
Correlation matrix for the a) 300 m and b) 1000 m residential address buffers, and c) 50 m trip-based buffer (values from −1 to + 1 are presented from dark red to dark green, respectively)

The analysis of residential greenspace and MVPA-minutes did not provide clear evidence of associations with greenspace at either the 300 m or 1000 m buffers (Table 3). Coefficients of the increase in MVPA were generally small, and confidence intervals included 0 in fully adjusted models for all greenspace metrics (Table 3). Of the covariates, only walk score in the NDVI model (300 m buffer) showed a clear positive trend (1.13 MVPA-minutes [95% CI: 0.03 to 2.23]) per 1-unit increase in walk scores in fully adjusted models (data not shown).

**Table 3:** Regression analysis results of residential greenspace and daily minutes of moderate to vigorous intensity steps (MVPA-minutes)

Model	Greenspace metric	Change in daily MVPA-minutes (95% CI) for a 10 percentage point increase in greenspace marker based on buffer around place of residence	
		300 m	1000 m
**Model 1**: unadjusted	NDVI	−0.71 (−2.21 to 0.78)	−1.10 (−2.53 to 0.33)
	TCD	− 0.42 (− 2.44 to 1.61)	− 0.63 (− 2.44 to 1.17)
	GLU	−0.89 (− 2.45 to 0.68)	− 1.43 (− 2.81 to − 0.04)
**Model 2**: model 1 + adjustment for age + sex + season + bluespace	NDVI	− 0.45 (− 1.84 to 0.94)	− 0.60 (− 1.88 to 0.69)
	TCD	− 0.13 (− 2.03 to 1.77)	− 0.42 (− 2.09 to 1.25)
	GLU	− 0.91 (− 2.47 to 0.64)	− 1.13 (− 2.52 to 0.25)
**Model 3**: model 2 + adjustment for walk score + car + dog + education + employment	NDVI	1.14 (− 0.41 to 2.70)	0.39 (− 1.09 to 1.86)
	TCD	0.27 (− 1.73 to 2.28)	− 0.59 (− 2.30 to 1.12)
	GLU	− 0.49 (− 2.16 to 1.17)	− 0.97 (− 2.40 to 0.47)

*n* = 4 study centres; *n* = 118 individuals

All average trip-based greenspace coefficients were positively associated with MET-minutes in the unadjusted and adjusted models. NDVI and TCD were most strongly related to MET-minutes, compared to GLU, with very similar coefficient values (10.41 [95% CI: 4.43 to 16.39] and 10.63 [95% CI: 4.96 to 16.30] additional MET-minutes per 10 percentage point increase, respectively). Although less precise, estimates of the absolute increase in MET-minutes for cycling trips were consistently higher than those for walking (Table 4). Select environmental covariates also were positively linked with MET-minutes across the greenspace models, particularly walk score and the presence of bluespace (data not shown).

**Table 4:** Regression analysis results of MET-minutes with trip-based greenspace for overall and activity-specific findings

Model	Greenspace metric	Change in MET-minutes (95% CI) per 10 percentage point increase in mean trip-greenspace (50 m buffer)		
		MET-minutes		
		Overall	Walking^**a**^	Cycling^**a**^
**Model 1**: unadjusted	NDVI	7.34 (2.25 to 12.44)	4.24 (2.57 to 5.91)	13.65 (6.23 to 21.07)
	TCD	9.16 (2.63 to 15.69)	6.34 (3.78 to 8.91)	23.91 (2.85 to 44.97)
	GLU	3.15 (0.12 to 6.17)	2.96 (0.60 to 5.32)	7.29 (− 2.94 to 17.53)
**Model 2**: model 1 + adjustment for age + sex + season + bluespace	NDVI	7.20 (2.39 to 12.01)	4.30 (2.83 to 5.77)	13.73 (5.83 to 21.67)
	TCD	8.56 (3.04 to 14.09)	5.89 (3.91 to 7.87)	23.32 (2.54 to 44.09)
	GLU	3.18 (− 0.01 to 6.37)	2.90 (0.40 to 5.39)	7.89 (− 2.70 to 18.48)
**Model 3**: model 2 + adjustment for walk score + residential greenspace + car + dog + education + employment + weekday + weather	NDVI	10.41 (4.43 to 16.39)	7.81 (4.12 to 11.50)	15.53 (8.60 to 22.45)
	TCD	10.63 (4.96 to 16.30)	8.10 (4.93 to 11.28)	22.79 (5.24 to 40.34)
	GLU	3.36 (0.00 to 6.72)	3.29 (0.27 to 6.30)	6.00 (− 3.34 to 15.34)

*n* = 3 study centres; *n* = 60 individuals; *n* = 1014 trips

^a^Adjusted for interaction between greenspace and activity

## Discussion

Proximity to greenspace, typically in a residential setting, has been associated with a host of positive health outcomes. In this study, we used objective indicators to explore greenspace and outdoor PA as a potential underlying mechanism for health. We found no evidence to suggest individuals who lived in greener neighbourhoods engaged in greater levels of MVPA than those residing in less green areas. On the other hand, we found strong support that individuals choose greener settings for physically active travel of higher intensity and/or longer duration.

### Residential greenspace

We found no clear evidence that the amount of greenspace around the home was associated with overall MVPA. A similar finding has been reported in some studies [53, 54] but not in others [23, 43], with some of the earlier work examining comparable residential greenspace metrics and objective PA, the majority of which examined GLU as the exposure of interest. The number of parks within a 1 km residential buffer, but not the residential distance to the nearest park, was associated with objective MVPA in a group of US adults [42]. Likewise, the number of parks within 500 m and 1 km buffers was also found to be the strongest indicator for MVPA minutes in an eight-country study; park area within those same buffer sizes (a metric similar to the GLU metric in the current study) did not indicate a correlation with PA [45]. Sallis et al. [43] also found parks within 500 m of residential addresses to be positively associated with objective MVPA, after adjusting for walkability features (also significant), in a large sample of individuals from 10 countries. A study examining GLU (i.e., parks and other green land uses) and objective MVPA in Dutch adults aged 45–65 years found positive results, but only with smaller buffers (25–400 m) [23]. Triguero-Mas et al. [53] found overall MVPA activity was not associated with GLU situated within 300 m of home addresses in European adults, but was associated with contact and exercise specifically in natural outdoor environments; researchers did not account for walkability. We identified only one previous study that examined residential NDVI, which found no statistical links with overall objectively measured MVPA, and an inverse relationship with MVPA within a 1 km home buffer, in a sample of adult trail users in the US, [54]. We are unaware of any previous studies that compare the amount of residential tree canopy to objective measures of PA.

While some studies have found positive correlations between residential greenspace and objective MVPA, albeit mainly with the number of nearby parks, the existing evidence is neither consistent nor comprehensive. Our study found a mix of positive and negative greenspace effects, which may have achieved statistical significance (in either direction) with a larger sample size. Sample size notwithstanding, there are several reasons that may explain the lack of stronger findings: walkability indicators have typically been shown to be as or more important than nearby greenspace [54] (identified in the current study), the physical environment may be less important to influence exercise in parents of young children [6], PA in nearby parks has been found to constitute a small proportion of overall PA [49], and perhaps most pertinent is that MVPA may have occurred outside the 300 m and 1000 m buffers employed in the present study. Most participants in our study owned a car; Hillsdon et al. [20] found that car owners engaged in more than 60% of outdoor PA outside of the neighbourhood, as defined by an 800 m residential buffer. Thus, the amount of greenspace within a residential area may not be as important for people with access to a vehicle.

### Path-based greenspace

In our analysis of trip-specific data, we found positive links between the amount of vegetation (NDVI) and tree coverage, and to a lesser degree GLU, with longer and more active journeys. Few studies have used a GPS approach to combine greenspace exposure with objective PA in adults, but all have found some indication of a positive trend with PA. James et al. [22] assessed momentary exposure to NDVI, as opposed to trip-level averages as analysed in the current study, in female nurses in the US and found a positive relationship with accelerometer counts per minute, particularly when walkability was low. A study of a similar design to that of James et al. recruited trail users in the US and found NDVI to be positively associated with a higher likelihood of MVPA [51]. Houston [21] used a land cover map (including greenspace as tree canopy, irrigated grass cover, or non-irrigated grass cover/bare soil) and identified significant positive associations with the likelihood of adults engaging in MVPA. The amount of GLU at trip origin and end was associated with a higher probability of walking in a study in France, which found that trip-level characteristics outweighed those of the residential environment [8]. A study of adults in Barcelona that also used the Moves app found both the proportion of large parks and tree density along routes to be positively associated with walking minutes [58].

We found higher effects of greenspace on cycling compared to walking, though the former had a wider range of possible effects. Few previous studies have examined greenspace with objective adult physical activity measures of both walking and cycling. Le et al. [31] quantified the built environment surrounding bicycle and pedestrian counters in 20 US cities and found a greater positive effect on cycling than walking (though greenspace and bluespace were combined in their analysis). Our results with objective measures support studies of self-reported cycling. Commuters in Barcelona were more likely to be cyclists with higher greenness in the study/work environment; interestingly, the greenness of the route was not significant, though commuting journeys were estimated by shortest distance rather than those actually travelled [11]. Questionnaire respondents in Stockholm reported greenery to be one of the most important factors to stimulate cycle commuting [60]. Although we looked at all active trips (i.e., not just those for commuting), our results build on this earlier research to suggest that greenness, through both overall vegetation and trees, might enhance and encourage all active transport by providing a more pleasant route.

### Overall findings

We examined both residential and active transport environments, which provided an opportunity to compare and contrast these exposures using the same dataset. We found no evidence to support the residential environment being associated with objective MVPA, though our analysis was based on steps and therefore would have only pertained to walking or running. This analysis also only related to the availability of greenspace, not necessarily its use. We also examined greenspace levels of the entire route for those trips involving walking or cycling. Whereas contemporaneous momentary designs (i.e., matching exposure and PA at points in time) are more likely to reveal typical behaviours in certain settings (e.g., less PA in commercial areas and more PA in natural areas, such as greenspaces) [9], our analysis took into account average characteristics of the entire route. Therefore, our approach was more equipped to answer the question: given an individual has decided to undertake PA, how is greenspace associated with the intensity and duration of activity? In other words, how does the presence of greenspace factor in the selection of environments through which individuals choose to travel or exercise? We found clear evidence indicating both NDVI and TCD as greenspace markers were positively linked to intensity and duration of activity, while adjusting for other characteristics of the built environment. Certain such characteristics, namely walk score, were consistently related to higher levels of PA; nevertheless, the different scales of greenspace markers and walk score render it difficult to identify which is the more influential factor for PA. We also found positive links to MVPA with the proportion of GLU along a route, but not specifically for cycling trips. The use of a particular greenspace for a specific activity, namely cycling in this case, may be more dependent on certain features, including size, cycling routes, and wooded areas, which were not quantified explicitly in the overall area-based GLU metric employed in our study [44]. In addition, the GLU map we used was based on a lower spatial resolution (100 m) than the NDVI (10 m) or TCD (20 m) metrics. Therefore, the use of this coarser resolution, with greater aggregation of features and potential exclusion of smaller parks, might help explain the weaker associations we observed between GLU and PA indicators [30].

### Strengths and limitations

Our study had several key strengths. We assessed the importance of both the residential and active route settings, thus developing dynamic and multi-contextual environmental exposures [29], with two objective MVPA indicators. We also used three different objective indicators to help characterise greenspace features of the built environment, with two different residential buffer sizes to help address the modifiable areal unit problem [21]. These advantages notwithstanding, there were some limitations to our research. Although we did not explicitly address reasons for choosing residential locations, we attempted to control for self-selection in the trip-based analysis by including residential greenspace levels and found our results to be unchanged. Several greenspace and PA studies have attempted to account for self-selection by including reasons for choosing to live in their neighbourhood (e.g., access to places that support PA, access to local services). Associations with PA have persisted after adjustment for such factors [24, 34]. Therefore, it is not likely that residential self-selection would have strongly biased our results. However, it would have been beneficial for our analysis, and understanding of the importance and role of greenspace, to know the purpose(s) of each trip.

While the Moves app has been shown to accurately provide location, speed, and duration, the software has had challenges identifying multi-modal trips, which may have been included as discrete events in our analysis [4]. In addition, there was a lower proportion of participants with complete Moves data than that provided by the Fitbit; this might be due to phones running out of batteries or being switched off. Our sample size was quite modest, and our study demographic was limited to parents of young children, which could restrict the generalisability of key findings. Although Candelaria et al. [5] found little difference in the amount of objective MVPA recorded between parents of young and older children and non-parents, the mean MVPA-minutes in our sample was much lower than Candelaria et al. and in studies with other demographics [25] (~ 12 vs > 30 mins/day). If MVPA steps were underestimated in our study, any association with residential greenspace levels might have been hindered. The majority of our study sample was university educated and owned a car, indicative of a higher SEP; lower SEP individuals might experience different relationships between greenspace and MVPA [17]. As noted above, the environments of study/work may be important, but we did not have this information for all study participants. We also were not able to distinguish whether study subjects were currently working or on maternity/paternity leave. We characterised surrounding streets and intersections using maps from 2019, though personal monitoring took place over 2015–2016; therefore, some misclassification of walkability may have been introduced by any road network changes occurring in the intervening years, but it is expected that any impact on our results would have been minimal. Each subject participated in only one personal monitoring period in the HEALS study; repeating data collection with participants during different times of the year may provide insights into the role of temporal/seasonal factors of greenspace and PA.

## Conclusion

We examined PA as a potential explanatory pathway for observed associations between health and greenspace, assessing both residential and trip-specific environments. We found little evidence to suggest residential greenspace was associated with higher levels of MVPA, regardless of where that may take place. On the other hand, we found clear, positive associations between intensity and duration of activities with the average amount of greenness and tree coverage along a route, which was true for both walking and even more so for cycling. We suggest future research to build on this proposed model of specific pathways by examining larger, more diverse populations, while also investigating the influence of greenspace for trip purpose and route preference.

## Supplementary Information

**Additional file 1.**

## Data Availability

Access to the personal data used in this study is governed by the HEALS Data Management Plan. The environmental datasets obtained in this study are publicly available from the specific references herein. The datasets generated and/or analysed during the current study are not publicly available due to reasons of ensuring anonymity for study subjects. Specific data may be made available from the corresponding author on reasonable request.

## Abbreviations

GLU: Green land use
HEALS: Health and Environment-wide Associations based on Large population Surveys
MET: Metabolic task equivalent
MVPA: Moderate to vigorous physical activity
NDVI: Normalised Difference Vegetation Index
PA: Physical activity
SEP: Socioeconomic position
SD: Standard deviation
TCD: Tree cover density
WHO: World Health Organization
