# Supplementary material for: Neighbourhood and path-based greenspace in three European countries: associations with objective physical activity
Source: BMC Public Health. 2021 Feb 4;21:282. doi: 10.1186/s12889-021-10259-0 (PMC7860634; doi:10.1186/s12889-021-10259-0)
Supplement: Supplementary file 1 — Additional file 1. [file 12889_2021_10259_MOESM1_ESM.docx]

**Supplementary Material**

Contents

[HEALS socioeconomic questionnaire 1](#_Toc61508091)

[HEALS household questionnaire 11](#_Toc61508092)

[Supplementary Tables 18](#_Toc61508093)

# HEALS socioeconomic questionnaire

**HEALS Pilot Study**

**Socioeconomic Status Questionnaire**

(To be administered by field staff to adult participant)

*********************************************************************

**HOUSEHOLD ID:**

**FIELD STAFF NAME:**

**QUESTIONNAIRE DATE:**

**QUESTIONNAIRE START TIME:**

**QUESTIONNAIRE END TIME:**

**INTERVIEW WITH: CHILD’S MOTHER** □_2_ **CHILD’S FATHER** □_1_

Reference period: _ _/201_ Respondent ID………

**In this study we are looking at the environment that children live in and how this may affect their health and wellbeing now and in the future. Thank you for taking part.**

**In this survey we are asking some questions about the people that you and your child live with. We have a particular focus on transport and type of work because this will lead to exposure to different chemicals.**

1. **HOUSEHOLD QUESTIONS**

**A1. Please tell us about everybody in your household?** [If age not known, please give best estimate] The main earner is the household member who usually earns the most money (Hh1ppl) (_MEMID)

| **Relationship to you**  **e.g. daughter/husband/partner/lodger/parent(p_rel)** | **Female** | **Male(gender)** | **Age(age)** | **Main earner(main earner)** | **Study child**  **(study child)** |
| --- | --- | --- | --- | --- | --- |
| 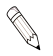 **Myself** | □_2_ | □_1_ | 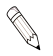 | □ | □ |
|  | □_2_ | □_1_ |  | □ | □ |
|  | □_2_ | □_1_ |  | □ | □ |
|  | □_2_ | □_1_ |  | □ | □ |
|  | □_2_ | □_1_ |  | □ | □ |
|  | □_2_ | □_1_ |  | □ | □ |
|  | □_2_ | □_1_ |  | □ | □ |
|  | □_2_ | □_1_ |  | □ | □ |
|  | □_2_ | □_1_ |  | □ | □ |
|  | □_2_ | □_1_ |  | □ | □ |

**A2. Do you have any children who do not currently live in your household?** (Hh2nliv_i and Hh2nliv_ii)

| No □_2_ | Yes □_1_ Please give ages : | 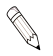 |
| --- | --- | --- |

**A3a. Please indicate your legal marital status:** (Hh3stas)

Married □_1_ Civil partnership □_2_ Single □_3_ Separated □_4_ Divorced □_5_ Widowed □_6_

| **A3b. If you ARE living with a spouse or partner in what year did you start living together?** (Hh4livt) | 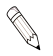 |
| --- | --- |

**A4a. Does your household include:** (Hh5inc)

both your child’s parents □_1_ one of your child’s parents (yourself) □_2_

**A4b. IF you are NOT living with the child’s other parent on average how often does your child usually spend time with him/her?** (Hh6time)

| 5 to 7 nights a week | □_5_ |  | less than once a fortnight | □_2_ |
| --- | --- | --- | --- | --- |
| 3 to 4 nights a week | □_4_ |  | does not see other parent | □_1_ |
| 1 to 2 nights a week or 1 night per fortnight | □_3_ |  |  |  |
|  |  |  |  |  |

**A5. Does your child’s other parent support your child financially nowadays?** (Hh7fin)

Regularly □_1_ Sometimes □_2_ Never □_3_

1. **FAMILY BACKGROUND**

**B1. Please tell us about the ethnicity of yourself and your child’s other parent (even if not living with you)** (Fb1eth)

| 1. **Ethnic group** | | | 1. **Religion** | | | 1. **Place of birth** | | |
| --- | --- | --- | --- | --- | --- | --- | --- | --- |
|  | **You (a_i)** | **Other parent (a_ii)** |  | **You (b_i)** | **Other parent (b_ii)** |  | **You (c_i)** | **Other parent (c_ii)** |
| White (Scottish/British) | □_1a_ | □_1b_ | No religion | □_1a_ | □_1b_ | Scotland | □_1a_ | □_1b_ |
| White (other) *(write in below)* | □_2a_ | □_2b_ | Buddhism | □_2a_ | □_2b_ | Rest of UK | □_2a_ | □_2b_ |
| Mixed *(write in below)* | □_3a_ | □_3b_ | Christian | □_3a_ | □_3b_ | Republic of Ireland | □_3a_ | □_3b_ |
| Arab | □_4a_ | □_4b_ | Hinduism | □_4a_ | □_4b_ | Poland | □_4a_ | □_4b_ |
| Asian (Pakistani, Indian,  Bangladeshi) | □_5a_ | □_5b_ | Jewish | □_5a_ | □_5b_ | India | □_5a_ | □_5b_ |
| Asian (Chinese, Japanese, Korean) | □_6a_ | □_6b_ | Muslim | □_6a_ | □_6b_ | Pakistan | □_6a_ | □_6b_ |
| Asian (other) *(write in below)* | □_7a_ | □_7b_ | Sikh | □_7a_ | □_7b_ | Germany | □_7a_ | □_7b_ |
| Black (African, Caribbean etc) | □_8a_ | □_8b_ | Other | □_8a_ | □_8a_ | *Other (write in below* | □_8b_ | □_8b_ |
| Other *(write in below)* | □_9a_ | □_9b_ |  |  |  |  |  |  |
| 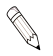 | | | 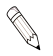 | | | 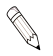 | | |

**B2. Does your household have a car/van for private use?** (Fb2car)

Yes (bought new within the last 6 months) □_1_ Yes (but not bought new within the last 6 months) □_2_

No - cannot afford a car □_3_ No - other reason □_4_

**B2a. If your household has one or more cars how often do the following people travel in it/them, in hours per day:** (Fb3crhr)

|  | **Weekday** | **Weekend day** |
| --- | --- | --- |
| Yourself (a_i = weekday, a_ii = weekend) | 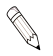 |  |
| Study child (b_i = weekday, b_ii = weekend) |  |  |
| Your partner (if applicable) (c_i = weekday, c_ii = weekend) |  |  |
| Main earner (if different) (d_i = weekday, d_ii = weekend) |  |  |

**B3. What is the highest level of successfully completed education for:** (Fb4ed**)**

|  | **You (a)** | **Other parent (b)** | **Main earner (if different) (c)** |
| --- | --- | --- | --- |
| None | □_1a_ | □_1b_ | □_1c_ |
| School | □_2a_ | □_2b_ | □_2c_ |
| Vocational/apprenticeship | □_3a_ | □_3b_ | □_3c_ |
| University/degree -level | □_4a_ | □_4b_ | □_4c_ |
| Other (write in) | □_5a_ | □_5b_ | □_5c_ |
|  | 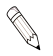 |  |  |

**B4. What is the current economic activity of:** (Fb5ecac)

|  | **You (a)** | **Other parent (b)** | **Main earner (if different) (c)** |
| --- | --- | --- | --- |
| Working for pay or profit (including unpaid work for a family business or holding; an apprenticeship or paid traineeship; currently on maternity, parental, sick leave or holidays) | □_1a_ | □_1b_ | □_1c_ |
| Pupil, student, further training, unpaid work experience | □_2a_ | □_2b_ | □_2c_ |
| In retirement (including early retirement) | □_3a_ | □_3b_ | □_3c_ |
| Permanently sick or disabled | □_4a_ | □_4b_ | □_4c_ |
| Caring for home and/or family (unpaid) | □_5a_ | □_5b_ | □_5c_ |
| Unemployed | □_6a_ | □_6b_ | □_6c_ |
| 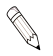Other (write in) | □_7a_ | □_7b_ | □_7c_ |

1. **OCCUPATION QUESTIONS**

**C1. Please tell us about the current (or most recent) job of yourself, your child’s other parent and main earner (if different)** (Oc1job) (**MEMID)**

|  | **Yourself (MEMID = 1)** | **Child’s other parent (MEMID = 2)** | **Main earner**  **(if different) (MEMID = 3)** |
| --- | --- | --- | --- |
| Does not work(Oc1job_a)  (if no one applicable works go to section D) | □_1a_ | □_1b_ | □_1c_ |
| Job title(Oc1job_b) | 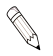 |  |  |
| Full time_1_ or part time_2_?(Oc1job_c) |  |  |  |
| Main job tasks(Oc1job_d) |  |  |  |
| Main activity of the employer/business(Oc1job_e) |  |  |  |
| Tick box if self employed(Oc1job_f) | □_2a_ | □_2b_ | □_2c_ |
| Number of supervisees/ employeesOc1job_g) |  |  |  |
| Number of people in company(Oc1job_h) |  |  |  |
| Number of hours usually worked per week?(Oc1job_i) |  |  |  |

| Usual transport to work (please tick the one for each person)(Oc1job_j) |  |
| --- | --- |

| Work mainly at or from home(1) | □_3a_ | □_3b_ | □_3c_ | □ | □ |
| --- | --- | --- | --- | --- | --- |
| A car or van(2) | □_4a_ | □_4b_ | □_4c_ | □ | □ |
| Bus(3) | □_5a_ | □_5b_ | □_5c_ | □ | □ |
| Train(4) | □_6a_ | □_6b_ | □_6c_ | □ | □ |
| Motorcycle, scooter or moped(5) | □_7a_ | □_7b_ | □_7c_ | □ | □ |
| Bicycle(6) | □_8a_ | □_8b_ | □_8c_ | □ | □ |
| On foot(7) | □_9a_ | □_9b_ | □_9c_ | □ | □ |
| Other means of transport(8) | □_10a_ | □_10b_ | □_10c_ | □ | □ |
| Don’t know(9) | □_11a_ | □_11b_ | □_11c_ | □ | □ |

1. **DAYCARE QUESTION**

**D1.** **Has your child/children EVER being looked after by other people than yourself?** (Dc1othr)

Yes □_1_ No □_2_

**D1a. If yes, can you tell us when they started and stopped (if applicable) and how many hours per week they usually spend with each?** (Dc2dchr)

| **(pID)** | **Date started**  **(month/year)** (a) | **Date Stopped**  **(month/year)**  **(if applicable)** (b) | **Days of the week** (c) | **Average number of hours per week**  **(include any changes in hours)** (d) | **If takes place outside your home then please give address** (e) |
| --- | --- | --- | --- | --- | --- |
| Child’s other parent(pID_1) | 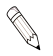 |  |  |  |  |
| Partner (if different from father)(pID_2) |  |  |  |  |  |
| Child's grandparent(s)(pID_3) |  |  |  |  |  |
| Child's older brother/sister(pID_4) |  |  |  |  |  |
| Another relative(pID_5) |  |  |  |  |  |
| A friend or neighbour(pID_6) |  |  |  |  |  |
| Nanny/carer in home(pID_7) |  |  |  |  |  |
| Childminder(pID_8) |  |  |  |  |  |
| Day nursery or crèchepID_9) |  |  |  |  |  |
| Special needs nursery(pID_10) |  |  |  |  |  |
| Playgroup, nursery school or pre-school(pID_11) |  |  |  |  |  |

1. **HISTORIC DATA**

We would like to know about the environment that important people in your child’s life have lived and worked in so we would like you to tell us about where you and your child’s other parent and grandparents have lived and worked throughout your lives. Don’t worry if you don’t know all the details – please just tell us the information that you do know.

**E1. Please list previous addresses – please be as precise as you can recall but if you do not know the address please give the name of the village/town and country** (Hi1add)

**E1a. Yourself** (Hi1add_pID=1) [for this entry, each address line should be added as an additional number under Hi1add_num, for example, if there are 3 entries in the table, then there should be 3 rows for Hi1add_pID=1, corresponding to Hi1add_num=1, 2, 3]

| **From (month and year)Hi1add_from** | **To (month and year)Hi1add_to** | **Address (as much information as known)Hi1add_add** |
| --- | --- | --- |
|  |  |  |
|  |  |  |
|  |  |  |
|  |  |  |
|  |  |  |
|  |  |  |
|  |  |  |

**E1b. Child’s other parent** (b) (Hi1add_pID=2)

| **From (month and year) Hi1add_from** | **To (month and year) Hi1add_to** | **Address (as much information as known) Hi1add_add** |
| --- | --- | --- |
|  |  |  |
|  |  |  |
|  |  |  |
|  |  |  |
|  |  |  |
|  |  |  |
|  |  |  |

**E2. Please list the employment history of important people in your child’s life** (Hi2emp)

**E2a. Yourself (Hi2emp_pID = 1)** [for this entry, each address line should be added as an additional number under Hi2emp_num, for example, if there are 3 entries in the table, then there should be 3 rows for Hi2_emp_pID=1, corresponding to Hi2emp_num=1, 2, 3]

| **From (month and year)(Hi2emp_from)** | **To (month and year)(Hi2emp_to)** | **Occupation(Hi2emp_occupation)** | **Address (if known)(Hi2emp_add)** | **Indoor or Outdoor Job?(Hi2emp_ind_out** | |
| --- | --- | --- | --- | --- | --- |
|  |  |  |  | **Largely Indoor**  **(1)** | **Largely Outdoor**  **(2)** |
|  |  |  |  |  |  |
|  |  |  |  |  |  |
|  |  |  |  |  |  |
|  |  |  |  |  |  |
|  |  |  |  |  |  |
|  |  |  |  |  |  |

**E2b. Child’s other parent (Hi2emp_pID = 2)**

| **From (month and year)** | **To (month and year)** | **Occupation** | **Address (if known)** | **Indoor or Outdoor Job?** | |
| --- | --- | --- | --- | --- | --- |
|  |  |  |  | **Largely Indoor** | **Largely Outdoor** |
|  |  |  |  |  |  |
|  |  |  |  |  |  |
|  |  |  |  |  |  |
|  |  |  |  |  |  |
|  |  |  |  |  |  |
|  |  |  |  |  |  |

**E2c. Main earner (if different) (Hi2emp_pID = 3)**

| **From (month and year)** | **To (month and year)** | **Occupation** | **Address (if known)** | **Indoor or Outdoor Job?** | |
| --- | --- | --- | --- | --- | --- |
|  |  |  |  | **Largely Indoor** | **Largely Outdoor** |
|  |  |  |  |  |  |
|  |  |  |  |  |  |
|  |  |  |  |  |  |
|  |  |  |  |  |  |
|  |  |  |  |  |  |
|  |  |  |  |  |  |

# HEALS household questionnaire

**HEALS Pilot Study**

**HOUSEHOLD QUESTIONNAIRE**

*********************************************************************

**HOUSEHOLD ID:**

**QUESTIONNAIRE DATE:**

**HOUSING CHARACTERISTICS**

*[****Interviewer say:*** *First I will ask you a few general questions about your home].*

1. How many years have you lived in your current home? (Answer in number of years) ___________

□ -888 = Don't know □ -999 = Refused

1. How old is your current home? (Answer in number of years) __________

□ -888 = Don't know □ -999 = Refused

1. Have there been any renovations made to this home since you have been living here?

□ 0 = No □ 1 = Yes □ -888 = Don’t know □ -999 = Refused

*[****Interviewer:*** *Ask #4-5 if response to #3 was “Yes”. Otherwise mark” Not applicable”]*

1. In what year(s) was/were renovations made to the home? ___________________

□ -777 = Not applicable □ -888 = Don’t know □ -999 = Refused

1. What kinds of renovations have been made to the home? (Tick all that apply)
2. □ 1 = Wall painting/new wallpaper
3. □ 1 = Ceiling
4. □ 1 =Floor repair/polishing/varnishing
5. □ 1 =Water/sewage system repair
6. □ 1 =Window or door repair/replacement
7. □ 1 =Insulation repair/replacement
8. □ 1 =Wall construction/removing
9. □ 1 =Heating/cooling system
10. □ 1 = Building an extension to home
11. □ 1 = Other (please specify: ______________________)
12. □ 1 = None
13. □ -777 = Not applicable
14. □ -888 = Don’t know
15. □ -999 = Refused
16. Has there ever been any water damage in your home?

□ 0 = No □ 1 = Yes □ -888 = Don't know □ -999 = Refused

*[Interviewer: if answer to 6 is “yes”, ask 7 and 8. Otherwise mark” Not applicable”]*

1. If yes, where? (Tick all that apply)

a) □ 1 = bathroom

b) □ 1 = child’s bedroom

c) □ 1 = living room

d) □ 1 = kitchen

e) □ 1 = in other rooms

f) □ -777 = Not applicable

g) □ -888 = Don’t know

h) □ -999 = Refused

1. When? __________ month _____________ year

□ -777 = Not applicable □ -888 = Don’t know □ -999 = Refused

1. Do you use the same source of water for drinking and cooking?

□ 0 = No □ 1 = Yes □ -888 = Don’t know □ -999 = Refused

*[Interviewer: if answer to 9 is “no”, ask 10 and 11. Otherwise mark” Not applicable”]*

1. What source do you use for drinking?

□ 1 = Tap, no home treatment

□ 2 = Tap, with home treatment (Specify: ____________________)

□ 3 = Bottled

□ 4 = Other (Specify: _____________)

□ -777 = Not applicable

□ -888 = Don’t know

□ -999 = Refused

1. What source do you use for cooking?

□ 1 = Tap

□ 2 = Tap, with home treatment (Specify: ____________________)

□ 3 = Bottled

□ 4 = Other (Specify: _____________)

□ -777 = Not applicable

□ -888 = Don’t know

□ -999 = Refused

1. Are there any smokers in the household? Please tick any which apply.
2. □ 1 = Father
3. □ 1 = Mother
4. □ 1 = Siblings
5. □ 1 = somebody else, Who? ______________________
6. □ -777 = Not applicable
7. □ -888 = Don’t know
8. □ -999 = Refused

*[Interviewer: if answer to 12 is 2-5, ask 13 and 14. Otherwise mark” Not applicable”]*

1. If there are smokers in the family, do they smoke

□ 1 = usually, indoors

□ 2 = usually outdoors (e.g. on the balcony)

□ 3 = always outdoors, including visitors

□ -777 = Not applicable

□ -888 = Don’t know

□ -999 = Refused

1. How many cigarettes per day are smoked indoors in your home? (Eg. Father 3, mother 2, sister 5 = 10 cigarettes in all)

□ 1 = none

□ 2 =1-5 cigarettes

□ 3 = 6-10 cigarettes

□ 4 = 11-15 cigarettes

□ 5 = 16-20 cigarettes

□ 6 = 21-30 cigarettes

□ 7 = more than 30 cigarettes

□ -777 = Not applicable

□ -888 = Don’t know

□ -999 = Refused

1. Do you currently have pets? Please tick any which apply.
2. 1 = no
3. 1 = dog
4. 1 = cat
5. 1 = birds
6. 1 = other animals, which? __________________________
7. □ -777 = Not applicable
8. □ -888 = Don’t know
9. □ -999 = Refused

**IN-HOME ROUTINES**

*[****Interviewer say****: Next, I will ask you about some of your household routines]*

1. During what months do you generally cool your home using air conditioning equipment? (Tick all that apply)
2. □ 1 =January-March
3. □ 1 =April-June
4. □ 1 =July-September
5. □ 1 =October-December
6. □ 1 = none
7. During what months do you generally heat your home? (Tick all that apply)
   1. □ 1 =January-March
   2. □ 1 =April-June
   3. □ 1 =July-September
   4. □ 1 =October-December
   5. □ 1 = none
8. What is the main heating system in your residence?

□ 1 = none

□ 2 = Central heating with radiators

□ 3 = Electrical heating

□ 4 = Under floor heating

□ 5 = Heating in the ceiling

□ 6 = Air circulating heating system

□ 7 = Fireplaces or ovens

□ 8 = Other (*Specify: _________________ )*

□ -888 = Don’t know

□ -999 = Refused

1. What fuel do you use to heat your home? (Tick all that apply)
2. □ 1 = Electricity
3. □ 1 = Gas
4. □ 1 = Liquid fuel
5. □ 1 = Wood burning stove/ fireplace
6. □ 1 = Other (*Specify: _________________ )*
7. □ 1 = none
8. □ -888 = Don't know
9. □ -999 = Refused
10. When the weather permits, how often do you open windows or doors for several hours a day?

□ 1 = Never

□ 2 = Less than once a month

□ 3 = About one to three times a month

□ 4 = About once a week

□ 5 = Several times a week

□ 6 = Every day

□ -888 = Don’t know

□ -999 = Refused

1. What type of material are your sofas and armchairs? (Check all that apply)
2. □ 1 =Leather
3. □ 1 =Upholstered with fabric cloth
4. □ 1 =Upholstered with vinyl material
5. □ 1 =Other (Specify: _____________________________)
6. □ -777 = Not applicable
7. □ -888 = Don’t know
8. □ -999 = Refused
9. Which best describes your family’s habit regarding wearing shoes in the home?

□ 1 = Shoes are taken off prior to entering the home

□ 2 = Shoes are taken off right away after entering the home

□ 3 = Shoes are taken off prior to entering certain rooms

□ 4 = Shoes are not routinely taken off while in the home

□ -777 = Not applicable

□ -888 = Don’t know

□ -999 = Refused

1. What kind of stove do you use in cooking? Please tick any which apply.

□ 1 = electrical stove

□ 2 = gas cooking

□ 3 = something else, ______________________________

□ -777 = Not applicable

□ -888 = Don’t know

□ -999 = Refused

1. Do you have a ventilation hood above the stove?

□ 0 = No

□ 1 = Yes

□ -777 = Not applicable

□ -888 = Don’t know

□ -999 = Refused

*[Interviewer: if answer to 24 is “yes”, ask 25]*

1. If you answered YES, do you use the hood when cooking?

□ 1 = regularly

□ 2 = every now and then

□ 3 = seldom or never

1. How many hours per day is the whole family away from home on a typical weekday?

□ 1 = 0-4 hours per day

□ 2 = 5-10 hours per day

□ 3 = 11-16 hours per day

□ 4 = Greater than 16 hours

□ 888 = Don't know □ 999 = Refused

1. How many hours per day is the whole family away from home on a typical weekend day?

□ 1 = 0-4 hours per day

□ 2 = 5-10 hours per day

□ 3 = 11-16 hours per day

□ 4 = Greater than 16 hours

□ 888 = Don't know □ 999 = Refused

# Supplementary Tables

**Table S1.** Dates (dd/mm/yyyy) of monitoring periods and NDVI images for all HEALS households (n=131)**.**

| **City** | **Monitoring period** | | **NDVI** |
| --- | --- | --- | --- |
|  | **Start** | **End** |  |
| Edinburgh | 23/07/2015 | 28/07/2015 | 27/06/2018 |
| Edinburgh | 01/09/2015 | 08/09/2015 | 27/06/2018 |
| Edinburgh | 19/08/2015 | 25/08/2015 | 27/06/2018 |
| Edinburgh | 05/08/2015 | 11/08/2015 | 27/06/2018 |
| Edinburgh | 04/08/2015 | 11/08/2015 | 27/06/2018 |
| Edinburgh | 17/08/2015 | 24/08/2015 | 27/06/2018 |
| Edinburgh | 11/08/2015 | 17/08/2015 | 27/06/2018 |
| Edinburgh | 21/09/2015 | 28/09/2015 | 27/06/2018 |
| Edinburgh | 13/08/2015 | 20/08/2015 | 27/06/2018 |
| Edinburgh | 17/09/2015 | 23/09/2015 | 27/06/2018 |
| Edinburgh | 02/10/2015 | 08/10/2015 | 27/06/2018 |
| Edinburgh | 07/10/2015 | 13/10/2015 | 27/06/2018 |
| Edinburgh | 29/10/2015 | 04/11/2015 | 27/06/2018 |
| Edinburgh | 16/10/2015 | 21/10/2015 | 27/06/2018 |
| Edinburgh | 04/11/2015 | 10/11/2015 | 27/06/2018 |
| Edinburgh | 17/11/2015 | 24/11/2015 | 27/06/2018 |
| Edinburgh | 26/11/2015 | 03/12/2015 | 27/06/2018 |
| Edinburgh | 13/11/2015 | 19/11/2015 | 27/06/2018 |
| Edinburgh | 07/01/2016 | 14/01/2016 | 27/06/2018 |
| Edinburgh | 11/01/2016 | 18/01/2016 | 27/06/2018 |
| Edinburgh | 25/01/2016 | 01/02/2016 | 27/06/2018 |
| Edinburgh | 15/01/2016 | 21/01/2016 | 27/06/2018 |
| Edinburgh | 22/01/2016 | 29/01/2016 | 27/06/2018 |
| Edinburgh | 08/02/2016 | 15/02/2016 | 27/06/2018 |
| Edinburgh | 29/01/2016 | 05/02/2016 | 27/06/2018 |
| Edinburgh | 27/01/2016 | 03/02/2016 | 27/06/2018 |
| Edinburgh | 17/02/2016 | 23/02/2016 | 27/06/2018 |
| Edinburgh | 05/02/2016 | 12/02/2016 | 27/06/2018 |
| Edinburgh | 12/02/2016 | 19/02/2016 | 27/06/2018 |
| Athens | 29/06/2015 | 06/07/2015 | 10/07/2016 |
| Athens | 30/06/2015 | 05/07/2015 | 10/07/2016 |
| Athens | 06/07/2015 | 13/07/2015 | 10/07/2016 |
| Athens | 07/07/2015 | 13/07/2015 | 10/07/2016 |
| Athens | 13/07/2015 | 20/07/2015 | 10/07/2016 |
| Athens | 14/07/2015 | 20/07/2015 | 10/07/2016 |
| Athens | 20/07/2015 | 26/07/2015 | 10/07/2016 |
| Athens | 22/07/2015 | 28/07/2015 | 10/07/2016 |
| Athens | 27/07/2015 | 03/08/2015 | 10/07/2016 |
| Athens | 28/07/2015 | 03/08/2015 | 10/07/2016 |
| Athens | 17/08/2015 | 26/08/2015 | 10/07/2016 |
| Athens | 28/08/2015 | 03/09/2015 | 10/07/2016 |
| Athens | 03/09/2015 | 10/09/2015 | 10/07/2016 |
| Athens | 04/09/2015 | 09/09/2015 | 10/07/2016 |
| Athens | 09/09/2015 | 15/09/2015 | 10/07/2016 |
| Athens | 11/09/2015 | 16/09/2015 | 10/07/2016 |
| Athens | 15/09/2015 | 20/09/2015 | 10/07/2016 |
| Athens | 16/09/2015 | 21/09/2015 | 10/07/2016 |
| Athens | 21/09/2015 | 29/09/2015 | 10/07/2016 |
| Athens | 29/09/2015 | 04/10/2015 | 10/07/2016 |
| Athens | 30/09/2015 | 06/10/2015 | 10/07/2016 |
| Athens | 06/10/2015 | 12/10/2015 | 10/07/2016 |
| Athens | 07/10/2015 | 13/10/2015 | 10/07/2016 |
| Athens | 13/10/2015 | 18/10/2015 | 10/07/2016 |
| Athens | 14/10/2015 | 21/10/2015 | 10/07/2016 |
| Thessaloniki | 05/12/2015 | 11/12/2015 | 13/07/2016 |
| Thessaloniki | 09/12/2015 | 16/12/2015 | 13/07/2016 |
| Thessaloniki | 15/12/2015 | 22/12/2015 | 13/07/2016 |
| Thessaloniki | 16/12/2015 | 24/12/2015 | 13/07/2016 |
| Thessaloniki | 14/01/2016 | 20/01/2016 | 13/07/2016 |
| Thessaloniki | 14/01/2016 | 20/01/2016 | 13/07/2016 |
| Thessaloniki | 11/04/2016 | 18/04/2016 | 13/07/2016 |
| Thessaloniki | 18/04/2016 | 25/04/2016 | 13/07/2016 |
| Thessaloniki | 18/04/2016 | 25/04/2016 | 13/07/2016 |
| Thessaloniki | 25/04/2016 | 04/05/2016 | 13/07/2016 |
| Thessaloniki | 25/04/2016 | 04/05/2016 | 13/07/2016 |
| Thessaloniki | 04/05/2016 | 10/05/2016 | 13/07/2016 |
| Thessaloniki | 04/05/2016 | 09/05/2016 | 13/07/2016 |
| Thessaloniki | 10/05/2016 | 16/05/2016 | 13/07/2016 |
| Thessaloniki | 10/05/2016 | 16/05/2016 | 13/07/2016 |
| Thessaloniki | 16/05/2016 | 23/05/2016 | 13/07/2016 |
| Thessaloniki | 16/05/2016 | 23/05/2016 | 13/07/2016 |
| Thessaloniki | 24/05/2016 | 30/05/2016 | 13/07/2016 |
| Thessaloniki | 23/05/2016 | 30/05/2016 | 13/07/2016 |
| Thessaloniki | 30/05/2016 | 06/06/2016 | 13/07/2016 |
| Thessaloniki | 30/05/2016 | 06/06/2016 | 13/07/2016 |
| Thessaloniki | 06/06/2016 | 13/06/2016 | 13/07/2016 |
| Thessaloniki | 07/06/2016 | 14/06/2016 | 13/07/2016 |
| Thessaloniki | 14/06/2016 | 23/06/2016 | 13/07/2016 |
| Thessaloniki | 15/06/2016 | 22/06/2016 | 13/07/2016 |
| Utrecht | 12/03/2015 | 17/03/2015 | 08/09/2016 |
| Utrecht | 17/03/2015 | 24/03/2015 | 08/09/2016 |
| Utrecht | 13/04/2015 | 21/04/2015 | 08/09/2016 |
| Utrecht | 17/04/2015 | 23/04/2015 | 08/09/2016 |
| Utrecht | 22/04/2015 | 29/04/2015 | 08/09/2016 |
| Utrecht | 01/05/2015 | 08/05/2015 | 08/09/2016 |
| Utrecht | 13/05/2015 | 20/05/2015 | 08/09/2016 |
| Utrecht | 15/05/2015 | 22/05/2015 | 08/09/2016 |
| Utrecht | 18/05/2015 | 25/05/2015 | 08/09/2016 |
| Utrecht | 19/05/2015 | 26/05/2015 | 08/09/2016 |
| Utrecht | 19/05/2015 | 26/05/2015 | 08/09/2016 |
| Utrecht | 27/05/2015 | 03/06/2015 | 08/09/2016 |
| Utrecht | 29/05/2015 | 05/06/2015 | 08/09/2016 |
| Utrecht | 02/06/2015 | 08/06/2015 | 08/09/2016 |
| Utrecht | 02/06/2015 | 09/06/2015 | 08/09/2016 |
| Utrecht | 03/06/2015 | 10/06/2015 | 08/09/2016 |
| Utrecht | 04/06/2015 | 11/06/2015 | 08/09/2016 |
| Utrecht | 10/06/2015 | 17/06/2015 | 08/09/2016 |
| Utrecht | 11/06/2015 | 18/06/2015 | 08/09/2016 |
| Utrecht | 15/06/2015 | 22/06/2015 | 08/09/2016 |
| Utrecht | 15/06/2015 | 23/06/2015 | 08/09/2016 |
| Utrecht | 19/06/2015 | 26/06/2015 | 08/09/2016 |
| Utrecht | 22/06/2015 | 29/06/2015 | 08/09/2016 |
| Utrecht | 23/06/2015 | 30/06/2015 | 08/09/2016 |
| Utrecht | 24/06/2015 | 30/06/2015 | 08/09/2016 |
| Utrecht | 30/06/2015 | 07/07/2015 | 08/09/2016 |
| Utrecht | 01/07/2015 | 07/07/2015 | 08/09/2016 |
| Utrecht | 03/07/2015 | 10/07/2015 | 08/09/2016 |
| Utrecht | 07/07/2015 | 14/07/2015 | 08/09/2016 |
| Utrecht | 10/07/2015 | 16/07/2015 | 08/09/2016 |
| Utrecht | 13/07/2015 | 20/07/2015 | 08/09/2016 |
| Utrecht | 14/07/2015 | 20/07/2015 | 08/09/2016 |
| Utrecht | 17/07/2015 | 23/07/2015 | 08/09/2016 |
| Utrecht | 17/07/2015 | 23/07/2015 | 08/09/2016 |
| Utrecht | 20/07/2015 | 29/07/2015 | 08/09/2016 |
| Utrecht | 21/07/2015 | 29/07/2015 | 08/09/2016 |
| Utrecht | 22/07/2015 | 29/07/2015 | 08/09/2016 |
| Utrecht | 24/07/2015 | 31/07/2015 | 08/09/2016 |
| Utrecht | 29/07/2015 | 04/08/2015 | 08/09/2016 |
| Utrecht | 29/07/2015 | 06/08/2015 | 08/09/2016 |
| Utrecht | 29/07/2015 | 05/08/2015 | 08/09/2016 |
| Utrecht | 31/07/2015 | 05/08/2015 | 08/09/2016 |
| Utrecht | 03/08/2015 | 11/08/2015 | 08/09/2016 |
| Utrecht | 13/08/2015 | 19/08/2015 | 08/09/2016 |
| Utrecht | 04/08/2015 | 11/08/2015 | 08/09/2016 |
| Utrecht | 05/08/2015 | 12/08/2015 | 08/09/2016 |
| Utrecht | 17/08/2015 | 24/08/2015 | 08/09/2016 |
| Utrecht | 12/08/2015 | 18/08/2015 | 08/09/2016 |
| Utrecht | 12/08/2015 | 19/08/2015 | 08/09/2016 |
| Utrecht | 19/08/2015 | 26/08/2015 | 08/09/2016 |
| Utrecht | 24/08/2015 | 31/08/2015 | 08/09/2016 |
| Utrecht | 26/08/2015 | 31/08/2015 | 08/09/2016 |

**Table S2**. The specific Metabolic Equivalent Task (MET) values assigned for individual trips, as presented by Ainsworth et al. (2011).

| **Code** | **METs** | **Category** | **Specific Activities** |
| --- | --- | --- | --- |
| 01010 | 4.0 | Bicycling | Bicycling, <10 mph, leisure, to work or for pleasure |
| 01018 | 3.5 | Bicycling | Bicycling, leisure, 5.5 mph |
| 01020 | 6.8 | Bicycling | Bicycling, 10-11.9 mph, leisure, slow, light effort |
| 01030 | 8.0 | Bicycling | Bicycling, 12-13.9 mph, leisure, moderate effort |
| 01040 | 10.0 | Bicycling | Bicycling, 14-15.9 mph, racing or leisure, fast, vigorous effort |
| 01050 | 12.0 | Bicycling | Bicycling, 16-19 mph, racing/not drafting |
| 01060 | 15.8 | Bicycling | Bicycling, > 20 mph, racing, not drafting |
| 17151 | 2.0 | Walking | Walking, less than 2.0 mph, level, strolling, very slow |
| 17170 | 3.0 | Walking | Walking, 2.5 mph, level, firm surface |
| 17180 | 3.3 | Walking | Walking, 2.5 mph, downhill |
| 17190 | 3.5 | Walking | Walking, 2.8 to 3.2 mph, level, moderate pace, firm surface |
| 17200 | 4.3 | Walking | Walking, 3.5 mph, level, brisk, firm surface, walking for exercise |
| 17200+ | 6.0 | Walking | Walking, 3.6 to 4.0 mph, uphill, 1 to 5% grade |
| 17210 | 5.3 | Walking | Walking, 2.9 to 3.5 mph, uphill, 1 to 5% grade |
| 17211 | 8.0 | Walking | Walking, 2.9 to 3.5 mph, uphill, 6% to 15% grade |
| 17220 | 5.0 | Walking | Walking, 4.0 mph, level, firm surface, very brisk pace |
| 17220+ | 7.0 | Walking | Walking, 4.1 to 4.4 mph, uphill, 1 to 5% grade |
| 17230+ | 8.0 | Walking | walking, 4.5 mph, uphill, 1% grade |
| '+' indicates the MET code was modified in the present study. | | | |
